# Uptake, Engagement and Acceptance, Barriers and Facilitators of a Text Messaging Intervention for Postnatal Care of Mother and Child in India—A Mixed Methods Feasibility Study

**DOI:** 10.3390/ijerph19158914

**Published:** 2022-07-22

**Authors:** Swetha Sampathkumar, Meenakshi Sankar, Sankar Ramasamy, Nivedita Sriram, Ponnusamy Saravanan, Uma Ram

**Affiliations:** 1 Division of Health Sciences, Warwick Medical School, Gibbet Hill, University of Warwick, Warwick, Coventry CV4 7AL, UK; swetha.sampathkumar@warwick.ac.uk; 2 Buddhi Consulting Ltd., Lower Hutt 5011, New Zealand; sankarmeenakshi1@gmail.com; 3 Education Review Office, Wellington 6140, New Zealand; sankarramasamy09@gmail.com; 4 UT South Western Medical School, Dallas, TX 75390, USA; nivi.sriram1@gmail.com; 5 Brown University, Providence, RI 02912, USA; 6 Academic Department of Diabetes, Endocrinology and Metabolism, George Eliot Hospital, Nuneaton CV10 7DJ, UK; 7 Seethapathy Clinic and Hospital, Chennai 600014, India

**Keywords:** maternal health, mHealth, SMS intervention, text messaging intervention, postnatal care

## Abstract

This study aimed to test the feasibility and to identify barriers and facilitators towards adherence of a text messaging intervention for postnatal care in India. Mixed methods research involving both quantitative and qualitative methods were used. A survey questionnaire for feasibility and focus group interviews to identify the barriers and facilitators to the intervention were conducted. The top three reasons for activation of service were: helped the new mother to understand the changes (95%); provided continuation of care (90%) and clarified conflicting information (89%). Over 90% read the messages daily. 80% were happy with the message frequency. About 75% shared the content with others. The main reasons for non-activation were: 30% had technical issues, 15% did not think it would be useful, 17% did not have time to activate and for 5%, husbands made the decision. These findings were triangulated through the qualitative focus groups. The main themes identified via the focus groups were: (1) reliable, current information; (2) issues and themes well aligned with new mothers’ needs and priorities; (3) expanded the repertoire of information sources available; and (4) high-quality accessible information. The satisfaction and trust rates were high. This technology may be useful for health information intervention in specific postnatal areas.

## 1. Introduction

The postnatal period is traditionally defined as the period of 42 days following childbirth and is considered as one of the most critical phases for the mothers as well as the newborn babies [1]. Women and the babies should receive appropriate care in the postnatal period. There is an increasing understanding that the impact of the postnatal care goes beyond this period, and women and babies will benefit from care even beyond the first year post-delivery [2]. Indeed, the American College of Obstetricians and Gynecologists (ACOG) guidelines now defines the postpartum period as the first 12 weeks after birth [3]. Despite the proven importance, postnatal care consistently has the lowest coverage of interventions [4], and there is need for better strategies to improve postnatal care globally [5]. In low- and middle-income countries (LMICs), inequity prevails in access to postnatal care services and varies significantly with socioeconomic status and with area of residence [6].

In India, data suggests that a planned delivery in a healthcare facility can reduce neonatal deaths [7]. However, the best outcomes were seen when delivery in a healthcare facility was followed up with postnatal care, suggesting that about one third of all neonatal deaths (~100,000/year) in India can be prevented by delivery at a healthcare facility along with appropriate postnatal care [8,9]. However, this has been difficult to implement due to the need for mothers with newborn babies to return to a healthcare facility. Postpartum depression is a common, increasing problem that many new mothers experience in India. A meta-analysis by Upadhyay et al. showed a pooled prevalence of 22% [10]. In addition, there is a need for debunking many cultural rituals, myths and misconceptions around pregnancy and childbirth in many parts of India [11,12].

At the time of discharge from hospital after delivery, new mothers are given routine discharge advice, regarding their own care, activity, diet, breast feeding, common neonatal problems, and review dates. Although this is supported by some written information, this is often not read in detail by the new mothers. The postnatal period is a significant period of change for the woman and there is a chance that this information is misunderstood or forgotten. Globally, some women have reported lack of confidence in their parenting role as well as a high occurrence of parental stress [13,14].

Mobile health is increasingly being used for providing supportive healthcare and improving preventive health initiatives. Short message service (SMS) or text messaging has been used to support healthy behavior change and health care delivery processes [15,16,17,18]. There is some evidence that higher satisfaction rates occur among women who received messages that are relevant during the antenatal and perinatal period [19]. A meta-analysis by Yadav et al. shows that, in LMICs, mobile health interventions can increase the utilisation of antenatal and postnatal care compared to standard care [20]. Text messaging interventions can be used to enhance self-efficacy (e.g., reminders, feedback on treatment success, etc.), to provide a form of social support (from peers and health professionals) and to establish social networks (support groups, peer-to-peer networks) [21]. These interventions, through increased self-efficacy [22,23] and support mechanisms [24,25,26], may influence healthy behavior and enhance self-management of chronic illnesses [27].

There is evidence that women who received telephone-based peer support in the postnatal period were less likely to have postnatal depression and lower mean EPDS (Edinburgh postnatal depression scale) scores [28]. About 20% of women in the first 2 weeks of postnatal care felt their needs were not met [29]. Some of the factors contributing to this include lack of rest, lack of consistent advice, too much information in a short period and a perception that midwives were too busy.

There was also a higher likelihood of women keeping the postnatal visit appointments when messaging was introduced in the context of prevention of mother-to-child transmission in HIV [30]. A review by Lavender et al. suggests [31] that telephone support may be a promising intervention but emphasized the need for more evidence. In addition, text messaging interventions are becoming highly suited and relevant after the COVID-19 pandemic due to the advantages of remote delivery [32]. A Canadian postpartum text message program achieved improved psychosocial outcomes for postpartum women [33].

On the other hand, in India, studies on testing mHealth interventions for postnatal care are limited [34,35]. To address the above issues, an innovative solution to provide ongoing follow-up service to new mothers at discharge was designed. A text messaging service was launched with tips and essential postnatal information to help new mothers. The service aimed for new mothers to receive high-quality, timely, user-friendly information that is appropriate, credible, and evidence-based for the postnatal period. Our aim was to test the feasibility and acceptability, and to identify the barriers and facilitators towards adherence of this intervention.

## 2. Materials and Methods

### 2.1. Study Design

A routine service development of providing text messages to inform postnatal care was implemented at the two units of a hospital in Chennai, India. A subsequent evaluation of the service was carried out through mixed methods research: (1) by quantitative analysis and a telephone survey of 10% of the users and non-users of the service, and (2) by focus group interviews of the users to explore the barriers and facilitators towards adherence of the intervention. Subsequently, by parallel track analyses method [36], results from both the quantitative survey and focus groups were brought together as thematic findings with qualitative data presented as vignettes to illustrate and/or strengthen the findings from the survey.

**Figure 1:**
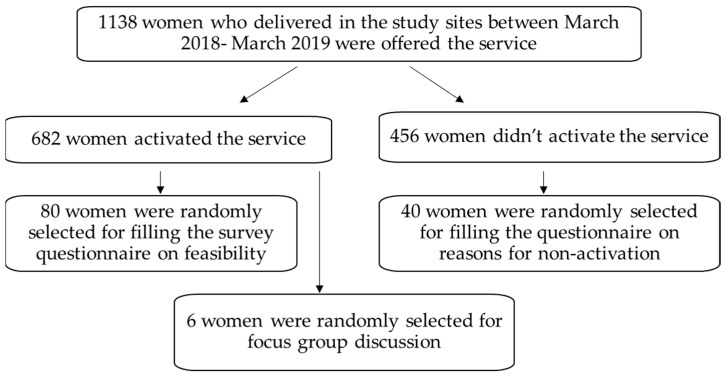
Study flow.

The study flow is represented in Figure 1.

A Theory of Change framework that provides an overview of how the elements of the service were intended to contribute to desired outcomes underpins the evaluation approach. A plausible theory of the change framework diagram was developed in discussions with key internal stakeholders (Supplementary Figure S1).

### 2.2. Text Messaging Service

Initially, the text messages were drafted by two consultant obstetricians working in collaboration with an expert skilled in writing content for health care mobile texting. The important areas that needed to be addressed were initially recorded and care was taken to ensure that all the key points in each area were covered. The texts were then organized in such a manner that messages would be delivered in a linear time-appropriate fashion, for example, postnatal review, vaccination reminders, return to normal physical activity post cesarean section, etc. (Supplementary Table S1). The structure and messages were then shared with five mothers to get their feedback. The intended frequency and changes suggested by the mothers and the obstetricians were incorporated to make the final list.

A small information leaflet regarding the postnatal messaging service was made available and given to all the mothers post-delivery. They were asked for their verbal consent to receive these messages as a free service and requested to call a number to activate the service (Supplementary Information S1 and S2). Once activated, they would receive one message every day for the next 100 days.

### 2.3. Participants

From March 2018 to March 2019, 1138 new mothers were offered the service. 60% (*n* = 682) had activated and engaged in the service. Women from both hospital sites were included in the survey and focus groups. The mothers were asked for their willingness to participate in the survey and verbal consent was taken to use their responses for research.

### 2.4. Data Collection

#### 2.4.1. Telephonic Survey

For the survey, a simple random sample of 120 women (10% of the total; 80 users and 40 non-users) was generated, matching their age, parity and mode of delivery. Data was collected through a telephone survey of users and non-users of the service. Basic demographic information on educational status, employment and family size was also collected from both groups of survey respondents. The information from both groups of respondents was intended to identify areas of improvement to the design and implementation of the service, thus, optimizing its value.

The users were asked about the reason for activation, the frequency, ease of understanding and readability of the messages, their satisfaction with the service and the whole service experience. They were also asked if they shared the messages with other members of family/friends and if they would recommend the same to family/friends. Finally, they were asked if they would pay for such a service. The non-users were mainly asked reasons for not activating the service. In the survey, the participants were asked to identify all that applied.

#### 2.4.2. Focus Group Discussions

In parallel, focus groups were conducted with users to dive deeper into respondent perceptions of the value of the service versus other information channels, such as the internet, peer support, some apps, and information from family/friends, etc. The facilitator was a woman with 2 decades of experience in conducting focus groups for other qualitative projects. The women selected for the focus groups (*n* = 6) were selected from the users who had not participated in the telephone survey. They were a mix of first-time mothers, second-time mothers and working and stay-at-home mothers. All mothers were in the 20–30 age group. Most had degree-level education and the rest had completed school and had the ability to understand messages in English. Thematic analysis was conducted for analysing the focus group data.

### 2.5. Statistical Analysis

Statistical analysis of the administrative data was undertaken to assess if there were any systematic differences between the users and non-users on key characteristics including socio-economic status. Binomial logistic regression was performed to assess the independent role of key characteristics on activating the service.

## 3. Results

### 3.1. Telephonic Survey

A total of 1138 new mothers were offered the service across the two units of the hospital between March 2018 to March 2019. Of them, 60% (*n* = 682) had activated and engaged in the service. A total of 120 women (80 users and 40 non-users) were randomly selected for the survey. A near 100% response rate was achieved as (1) the participants were explained about the study and recruited just before discharge, (2) the team introduced themselves that they are calling from the healthcare facility where they delivered, checked for a convenient time and called back. If there was no response, messages (voice and text) were left and followed up on a different date and time. Some calls were made after 5 pm and over the weekend to facilitate the completion of the survey.

The characteristics of the users and non-users are shown in Table 1. There were minor differences in participant characteristics between the users and the non-users. Users were more likely to be in the age group 25–35; more likely to have had one child, normal delivery and gestational diabetes mellitus (GDM). However, tests of significance showed significant differences in type of delivery between the two groups (*p*-value = 0.014). No significant differences were observed in other characteristics between the two groups (Table 1).

### 3.2. Reasons for Engaging in the Service

There were multiple reasons why users had chosen to use the service in the first instance (Table 2). The participants were asked to identify all that applied. The text messaging was a trusted reliable source of information, and the ability it provided to understand and initiate actions on their own were key to why the users chose the service.

### 3.3. Themes Identified from the Focus Group Discussion

The focus groups revealed that new mothers’ perceptions of relevance and value was derived from the following features of the service:

- Reliable, current, up-to-date information: Respondents believed the messages were crafted by the providers at the hospital and their knowledge and expertise lent credibility to the service.
- Topical issues and themes that are well aligned with new mothers’ needs and priorities: The messages covered a wide range of topics from breastfeeding to sleeping to even self-care for the new mother. The alignment of the topics to the development stage of the newborn was particularly valued by new mothers. Coverage of socially taboo topics, such as when sexual intercourse could begin or contraception, were seen particularly valuable as these topics could not be discussed with anyone other than your provider.
- Expanded the repertoire of information sources available to the new mother: New mothers have access to a wide range of information sources including the internet, social media, a variety of apps, books and magazines. Although this was valued, it was often challenging to ascertain the credibility of the information and to separate fact from fiction. The text messages by contrast had the seal of approval from ‘their’ provider and were tailored to meet their unique needs.
- High quality information in an accessible format: The messages were clear, and easy to read and understand. They were written in simple, plain English and were of an appropriate length. Receiving the information in a text format was particularly appreciated as mothers could read it repeatedly and save it for future use.

### 3.4. Value and Benefits of the Service

Identifying the outcomes achieved by this text messaging service is critical for informing future decisions, including potentially expanding the service to beyond the first 100 days or to other cohorts of pregnant women, i.e., those with GDM. The findings from the survey and the focus groups provide useful information in this regard. The outcomes achieved are summarized below: the text messaging service was described as highly relevant and offered great value to new mothers. The survey results showed (Table 3) that for most mothers, the service directly helped them to understand their situation and provide important health information:

- Increased new mothers’ awareness and understanding about postnatal healthcare practices: The messages provide an explanation for the issues experienced by new mothers; thus, they can be used to normalize the issues and provide clear actions to address the issues being faced. This helps to grow the mothers’ confidence in caring for her newborn which in turn improves the physical and emotional well-being of both the mother and the child.
- New mothers’ felt increased connectedness and emotional support as a direct result of the service: Many of the women interviewed talked about the helplessness, anxiety and an emotional low they experienced immediately following the birth of the child. Being home on their own with their newborn was a stressful time for many and there was a lack of connectedness with their usual social groups. The text messages helped overcome some of this loneliness and anxiety—it was a crutch and a link to the hospital, and this provided tremendous reassurance.

“*Post-delivery it is quite common to feel a bit low. When I went home and I received the first message, I felt happy. My heart lifted and I felt like I was being looked after even after leaving the hospital. I cannot express how I felt at the time*.”

(Mother, aged 28)

“*The vaccination reminders are so useful. I feel like someone cares about me and my child. I am not alone in this*.”

(Mother, aged 30)

- Prevented continuation of harmful and dated practices: All those interviewed talked about the ongoing stream of advice from well-wishers relating to the dos and don’ts associated with being a new mother. These included advice on appropriate diet (e.g., mangoes, ice cream, tender coconut water, spices, meat, egg); physical movement (e.g., no running or exercise); and outdated parenting tips (e.g., treating colic, feeding schedules, dealing with crying, sleeping). New mothers realized that these tips and practices were outdated and not supported by their providers. The text messages helped new mothers counter the pressure they felt from elder family members and break the cycle of continuing dated practices.

“*There are so many people who are quick to give advice. They may have had their child 50 years ago, but they still try to influence you, particularly when it comes to food. My mother-in-law for example tells me not to eat meat or eggs or even lentils. But in the hospital, the day after delivery they served me sambhar (lentil stew) and rice*.”

(Mother, aged 32)

“*I follow the advice given by the provider and the text message, which is also from the provider. They know best, they have seen us through the entire pregnancy, and they have seen so many pregnancies. Their knowledge is current. I trust them. I won’t follow the advice given by my mother or mother-in-law as it is not current. There is no scientific basis for what they say or ask you to do. They just scare you*.”

(Mother, aged 28)

- Improved quality of relationship between health practitioners and service users: The text messaging service made the users more alert to the current issues (common postnatal issues related to breastfeeding, sleep newborn care and common postpartum complaints related to bleeding and breast engorgement, etc.) faced by new mothers and built their confidence to deal with them. As a result, the mothers felt they did not need to ring the pediatrician for every little problem or concern, and they could wait and raise questions during the periodic visits. The proactive identification of issues and heightened vigilance led to improved self-efficacy.
- Positioning the healthcare facility as one that cares: Those interviewed were aware that the hospital was the only one providing this kind of text messaging service. Although this was not the reason for choosing this healthcare facility for their delivery, it certainly reinforced their view that this was the hospital which cared for its patients. The text messaging service extended their contact with the hospital and reassured them that the hospital cared for them even after they were no longer in the care of the hospital.

“*After you go home, you really feel alone. You are trained and equipped for delivery but after that you are left in the dark. But the texts are a gentle reminder that you are not alone. The hospital still cares for you, and I really feel good about that*.”

(Mother, aged 30)

### 3.5. Reflections on Process Measures

The design and implementation of the service is central to its success. The technological platform needs to work seamlessly in order to deliver value and minimize any frustration that users may feel when engaging with new technology. In this regard, the text messaging service was effectively implemented. Although overall the service was implemented well, there were some issues raised that merit further attention:

1. Timing: Information and invitation to sign up to the service was often provided at the time of discharge. This was a stressful period and new mothers reported feeling overwhelmed and distracted. They have limited ability to take in new information. There may be an opportunity to introduce the concept of the text messaging service when they come for their appointments leading up to the delivery, so that the pregnant woman is less pressured and is more likely to engage in such discussions.

“*At the hospital there are so many papers I had to sign. I don’t remember what I signed. I may have signed over all my assets away for all I know! This is not the right time for giving information about this useful service as I am not likely to remember anything*.”

(Mother, aged 27)

2. Repetitive: Some new mothers noticed that they frequently received the same message twice. This caused some frustration and needs to be addressed.
3. Clarity: For a few instances, the messages were contradictory to the information provided at the hospital. The demand feed versus scheduled feed was identified as one such example, where the hospital at the time of discharge advised that mothers should feed on a schedule; however, the text message suggested demand feed. This was confusing.
4. Alignment: Aligning the message to where the new mother is at in her first 100 days needs to be considered. The messages began from the date the service was activated. The service considered the day of activation as day 1. However, the activation may occur one week after the delivery which means the messages were not synchronizing with the development cycle of the newborn. There has to be a way of ensuring the messages are aligned to the delivery date and to make sure that the messages are synchronized with the baby’s evolution.
5. Miscellaneous: In some instances, when signing up for the service, the husband provided his phone number to activate the service. As a result, the messages went to him, and the mother missed out on direct access to the message.

### 3.6. Barriers for Non-Engagement

With reference to survey results, testing was undertaken to see if the proportions of users (*n* = 80) and non-users (*n* = 40) differed in their level of education (bachelors and above versus below), employment status (employed or not) and family type (joint vs. nuclear). Pearson’s Chi^2^ test showed no significant differences between the groups.

Overall, the survey results showed that the main reason for non-activation was lack of time to activate (18%). The other reasons were lack of understanding of the service (15%) and being unsure/don’t know why (13%). Only about 3% thought the service would not be useful. Half of the respondents provided other reasons among which the key ones were, gave a missed call but did not get activated, or stopped receiving messages after a few and husband made the decision.

A binomial logistic regression analysis was undertaken on the total study population to assess if specific characteristics of the mothers may have increased the probability of activating the service. At 95% confidence level, having a normal delivery was found to have an effect with an Odds Ratio: 1.341, 95% CI: 1.050–1.713, *p*-value: 0.019. Thus, the model suggests that women who had normal delivery have 1.34 times higher odds of activating the service than those women who had C-Section delivery (Supplementary Table S2).

## 4. Discussion

Our study looks at text messaging as a means of communication and support in the immediate postnatal period. Majority of users signed up because they thought that they would benefit from the additional support. The fact that some of the non-users did try to activate but could not for technical reasons suggests that if this gets resolved, more than 60% would use the service, as they actually thought this would be a value add to them.

Studies on testing mHealth interventions for postnatal care in India is limited [34,35]. And our study results indicate that new mothers who used the service found that the intervention to be informative, timely and empowering. In addition, an overwhelming majority reiterated the need for such a service. More importantly, it helped the mother feel connected with the health care provider and reduced their feeling of isolation. The text messaging service served as a crutch and a link to the hospital which provided then with tremendous reassurance. There was concordance between the quantitative and qualitative aspects of the study on all of these points. In most situations, the focus group discussions were also complimentary by providing additional, valuable insights on why the intervention was useful to new mothers.

Studies have looked at how mothers are underprepared for the first year of childbirth and how new mothers are coping with the paradox of being happy yet unhappy [37,38]. Access to reliable information at this stage can be critical in supporting mothers. Giving information at the time of leaving the hospital is not appropriate as it is a stressful time for mothers, and this has also come through in our study. These new mothers would benefit from appropriate information regarding postpartum care when they are going through the postnatal period. Technology-based solutions offer a means of doing this and also tailoring the message to where the mother is in her journey and providing her with the information when she needs it.

Applying the Theory of Change as a framework to map the outcomes also shows that the service acts as an important lever for supporting new mothers and increases their self-efficacy. Such a service offers new mothers with reliable information on topical issues that are well aligned with a new mothers’ needs, contributes to strengthening the relationship between service users and health professionals and deters use of outdated practices particularly relating to diet and exercise.

The findings also highlight some of the technical issues that need to be addressed to improve the uptake and delivery of the intervention. Some of the non-users cite either trying to activate or recalling that messages stopped after a while. This was curious as according to information from the service provider, they did not activate the service. This suggests that there may be technical issues for further exploration (e.g., interface of various phone devices with the messaging service, use of menu options) which prevented some of the women who wanted to activate the service but were not able to.

Although smartphone and internet penetration is high in India, the reasons for use are more about social connectedness and entertainment rather than as a source of education and information services [39]. The non-activation may be seen in the context of this underlying general trend.

### 4.1. Implications for Future Research

One of the important insights gained from this evaluation relates to when the new mother was invited to sign up for the text messaging service. The feedback from new mothers indicated that provision of information about the service, especially during the discharge process, is not the most appropriate time. In their view, it would be better to introduce the concept during the late third trimester antenatal visits, when there is more time to discuss and understand its value. This would mean the scene is set for activation to occur soon after delivery. It would also ensure that the messages remain relevant to the time in days from delivery as otherwise the date of activation is taken as day 1.

Other than the mode of delivery, there were no other significant differences between the observable characteristics of both groups. This implies that the study population is relatively homogenous and may require minimal targeting differentiation. One plausible explanation for why those who had a normal delivery were more likely to activate the service, is that those having a normal delivery are in a better state of wellbeing to take in additional information conveyed at the time of discharge and to act on it after reaching home. This reiterates the point that informing about this service at a time when all mothers have the time to understand the likely benefits of the service is important.

Many had expressed interest to receive such messages albeit at a reduced frequency beyond the 100 days. This could be particularly useful in high-risk situations such as GDM and pre-eclampsia where we could potentially address postnatal diet, exercise, and remind to test glucose levels and blood pressure. Since women felt the messages sent helped them get appropriate information with regard to diet and exercise, it could be expanded to address postnatal interventions in GDM, such as weight loss and postnatal screening for cardiometabolic disorders.

Given that text messaging is possible in the vernacular language, it may be possible to consider expanding the language options to reach a large number of women, particularly women who access public health care systems. These women do not have access to more sophisticated pregnancy information sources. Of course, the scalability of this has to be tested.

Exploratory work using behavioral insights (this is the use of empirical human behavior theories from the field of sociology and economics, such as the use of nudge theory of behavior change) [40,41] may reveal subtle differences in unobservable health-related behavioral characteristics of the study population. Some of these characteristics include care seeking, debunking myths, practicing safe practices as opposed to harmful cultural practices, problem solving, positive coping, etc. [42]. More importantly, such an approach may be better able to help frame the service that nudges positive behavior. This could lead to fine tuning the communications overall in terms of using a script while offering this service.

### 4.2. Study Limitations

Although our study was one of the first to provide a remote intervention through text messages in the postnatal period in India, which is co-developed with women, it had the following limitations. Firstly, our study did not explore whether the text message was understood and acted upon appropriately by the mothers as intended by the healthcare provider. This was outside the scope of this feasibility study. Secondly, we could not conduct the focus group among the non-users. However, the focus group among users highlighted that these messages were well received and acted on. Thirdly, there was no comparison to other modes of communication (e.g., websites) and so we cannot compare the effectiveness of this type of intervention (text message) with other forms of remote interventions. Finally, the results of the study cannot be generalized to a wider population as all the participants are from within a tier 1 Indian metropolitan city with the ability to understand text messages in English.

## 5. Conclusions

As a free service from the healthcare provider, this intervention shows that it can be a useful and effective way of reaching and providing new mothers with evidence-based health information during the postnatal stage. Thus, this evaluation adds to the existing evidence based on the relevance and usefulness of this mode of communication and in this instance of reaching a specific group namely, new mothers.

## Figures and Tables

**Table 1:** Characteristics of the study participants.

	Total (*n* = 1138)	Users (*n*= 682)	Non-Users (*n*= 456)
Age *n* (%)			
24 and less	159 (14.0)	87(12.8)	72 (15.8)
25 to 35	924 (81.2)	565(82.8)	359 (78.7)
36 and above	55(4.8)	30(4.4)	25 (5.5)
Parity *n* (%)			
1	635 (55.8)	389(57.0)	246(53.9)
2	395 (34.7)	236(34.6)	159(34.9)
3	79 (6.9)	40(5.9)	39(8.5)
4	21 (1.9)	13(1.9)	8(1.8)
5–7	8 (0.7)	4(0.6)	4(0.9)
Complication *n* (%)			
GDM	279 (24.5)	175(25.7)	104(22.8)
Hypothyroid	136 (11.95)	88(12.9)	48(10.5)
Others (Multiple)	108 (9.5)	56(8.2)	52(11.4)
None	615 (54.05)	363(53.2)	252(55.3)
Delivery type *n* (%)			
LSCS	469 (41.2)	261(38.3)	208(45.6)
Normal Delivery *	669 (58.8)	421(61.7)	248(54.4)

* *p*-value < 0.05.

**Table 2:** Reasons for engaging in the service (*n* = 80).

Why Did You Activate and Take Up This Service?		
It was a service offered by the hospital.	53.16%	42
It would help me understand what is going on with me and my child.	73.75%	59
I expected the information to be reliable.	29.49%	23
Such a service would help clarify any doubts I had after I went home.	42.31%	33
Having such information would help build my confidence in caring for my newborn.	35.00%	28
The information comes to me directly from the hospital.	25.00%	20
I would know first-hand what to do and take appropriate action.	21.05%	16
Any other please specify.	15.79%	3

**Table 3:** Value and benefits of the service (*n* = 80).

Which of the Following Statements Best Describe Your Experience of the Service?		
The texts helped me to understand my situation and what I was going through.	94.94%	75
It helped me to seek the right type of help at the right time.	82.28%	65
I was able to handle many issues myself.	68.35%	54
I felt the hospital continued to care for me even after I was discharged.	89.87%	71
It helped me to clarify conflicting information on topics like diet/activity/breastfeeding.	88.61%	70
It was information I could store and go back and read.	68.35%	54
It reminded me about vaccination and review visits.	78.21%	61
It addressed issues such as activity and contraception that I could then discuss with my provider.	50.00%	37

## Data Availability

Not applicable.

## Supplementary Materials

The following supporting information can be downloaded at: https://www.mdpi.com/article/10.3390/ijerph19158914/s1, Figure S1: The proposed theory of change; Table S1: Example of text messages; Table S2: Logistic regression results; Supplementary Information S1: Information and Informed Consent Sheet; Supplementary Information S2: Survey Questionnaire Templates.
