# Supplementary material for: Uptake, Engagement and Acceptance, Barriers and Facilitators of a Text Messaging Intervention for Postnatal Care of Mother and Child in India—A Mixed Methods Feasibility Study
Source: Int J Environ Res Public Health. 2022 Jul 22;19(15):8914. doi: 10.3390/ijerph19158914 (PMC9329952; doi:10.3390/ijerph19158914)
Supplement: Supplementary file 1 [file ijerph-19-08914-s001.zip › ijerph-1683082-supplementary.pdf]

International Journal of
Environmental Research and Public Health

MDPI

Supplementary Materials

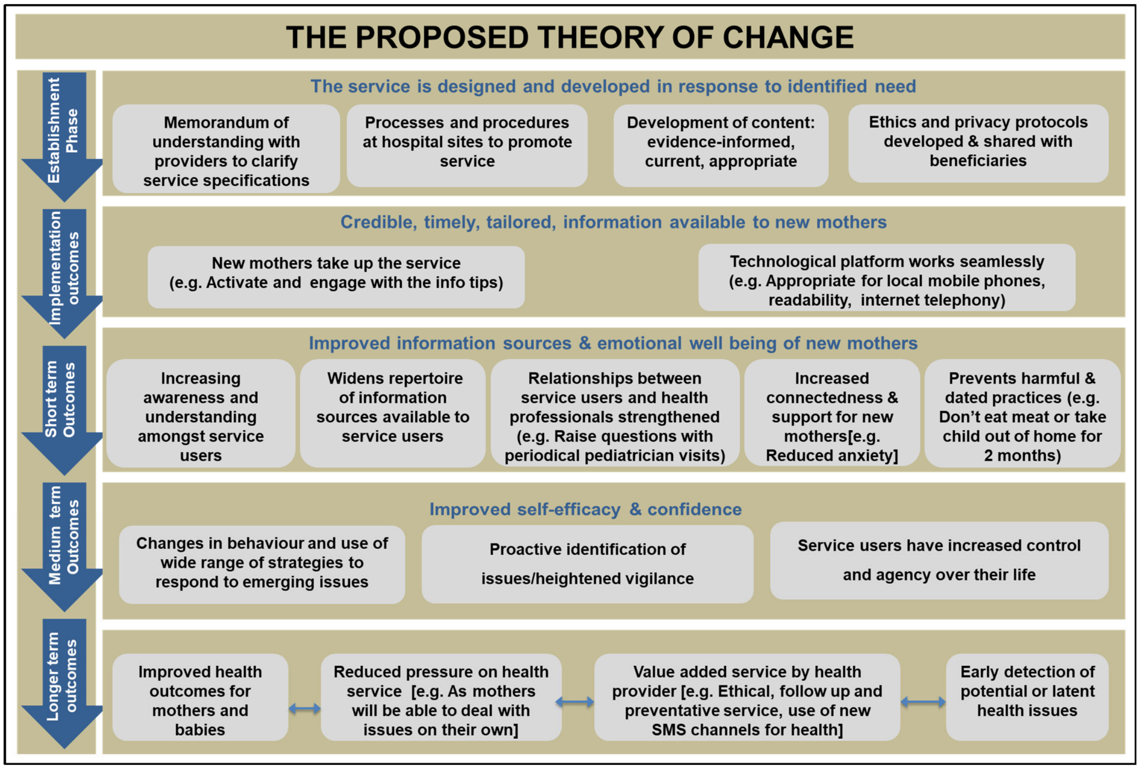

Supplementary Figure S1. The proposed theory of change.

Supplementary Table S1. Example of text messages.

1.	Best wishes as you being your journey with the little bundle of joy. PLEASE CALL OUR HOTLINE NUMBER AT ************
2.	Follow the hygiene measures advised to care for your c-section wound and/or episiotomy. If you experience heavy bleeding, fever or extreme pain, call the hospital ASAP.
3.	Breastfeeding is the best option for your baby. If you are unable to feed your baby/your milk is inadequate, you can supplement after seeking advice.
4.	Always wash your hands before breast feeding or handling the baby in any way. Wipe the nipples with a clean moist cloth before feeding.
5.	Babies can take from 8 to 12 feeds per day and this is best on demand. Make sure you also get some rest between feeds along with your baby.
6.	Dear Mother: please make/check your scheduled 2-week appointment. Make a note of what you would like to share with your doctor.
7.	Sometimes the breasts get very hard and painful. Feed the baby more often and use hot packs to relieve the pain and get milk moving again.
8.	Please hydrate properly. Also, adding adequate protein in your diet is important.
9.	Vegetables and fruits give us the necessary vitamins that we need. They also provide the fiber needed to make our stools soft.
10.	The green leafy vegetables provide a good source of iron and are available everywhere. Make sure to include them in your diet.
11.	Avoid too much ghee and high calorie food as it can result in weight gain and does not add nutrition to your diet.

12.	Lack of sleep can make new mothers upset and depressed. If this is happening do reach out to your doctor and discuss it.
13.	Your c-section sutures and vaginal area should have healed. If you are still experiencing pain, oozing or discharge, let your doctor know immediately.
14.	You should practice contracting and releasing the pelvic floor muscles at least 10 times a day to tighten your vaginal and pelvic area
15.	You can start lifting weights or doing more strenuous exercise by the 6th week after delivery. Please ensure your doctor has Ok'd especially for c-section.
16.	Your baby may be actually giving you a social smile. Your baby will recognize your face and your voice and respond. This is pure joy!
17.	Immunization helps prevent diseases like diarrhea, polio, liver disease, tetanus, typhoid, measles, mumps, rubella and some throat and chest infections.
18.	Dear Parents, you should also make time for each other after the birth of your new baby.
19.	You should be talking about the various options for contraception as a couple with your doctor before you start intercourse.
20.	If you have had gestational diabetes or blood pressure during pregnancy, we hope you have had a review with the doctors for this post-delivery

Supplementary Information S1: Information and Informed Consent Sheet

INTRODUCTORY BLURB

Hi, my name is XXXXX. I am calling on behalf of Seethapathy Hospital. We are undertaking a research project to find out about the text messaging service that was offered by the hospital to women who delivered their baby with the hospital from March 2018 to March 2019.

Our records show that (a) you registered for the Service but did not activate it OR (b) you registered and activated this Service.

We are interested in asking you a few questions about your opinions about this Service to help us improve the quality of this Service. Would you willing to answer a few questions about this Service?

Yes/No

If no, then ask:

What are your reasons for **not** wanting to participate in this survey?

____

Additional information

- The analysis will not identify you individually (directly or indirectly) and all findings will be treated in confidential.
    - Your name will not appear in any of the final analysis.
    - Your individual response will not be shared with anyone.
- The data will be held securely by Seethapathy Hospital and only accessed by those involved in the research.
- The data will be destroyed after the completion of the research.

Would you be prepared to participate in this survey now?

Yes/No

If no, then thank them for their time and end the call.

If they say yes, then proceed with the survey. Begin by informing them of the confidentiality clauses outlined in the box above.

Informed consent

Can you give me your consent for proceeding with this interview?

READ THE STATEMENT

I fully understand why Seethapathy Hospital is undertaking this research and agree to participate in this research.

Verbal consent Yes/No [Circle their response]

Supplementary Information S2: Survey Questionnaire Templates

TEXT MESSAGING SERVICE
SURVEY QUESTIONNAIRE—Non-Users

Fill these details prior to beginning the interview

Name		Serial number		Interviewer		Activated Y/N	

Thank you for agreeing to take part in this survey.

Those who said 'N' above i.e., did not activate by giving missed call.

1. Our records who that you registered but did not activate the service. Please tell me your reasons why you did not activate the service.
   [Tick all those that apply]

I did not fully understand the nature of the Service	
I did not have time to activate	
I did not think it would be useful	
I do not usually trust the information that comes on SMS	
I was concerned about data privacy	
I was not sure who was developing the content for the messages	
Don't know/not sure	
Any other/please specific	

Finish interview. Collect basic demographic information

2. Educational qualification:

Did not complete schooling	
Completed schooling	
Bachelor's Degree	
Above Bachelor's degree	
Any other?	

3. Are you employed? [Circle the correct response]

Yes	If yes, what is your current role?
No	

4. Which of the following describes your family unit?

Joint family [living with in-laws and/or the wider family]	
Nuclear family [husband/wife and their children]	
Any other	

TEXT MESSAGING SERVICE
SURVEY QUESTIONNAIRE—Users

Fill these details prior to beginning the interview

Name		Serial number		Interviewer		Activated Y/N	

Thank you for agreeing to take part in this survey.

Those who said 'Y' above i.e., activated the service by giving missed call.

Motivation to take up service

1. Why did you activate and take up this service? [Tick all that apply]

It was a service offered by the hospital	
It would help me understand what is going on with me and my child	
I expected the information to be reliable	
Such a service would help clarify any doubts I had after I went home	
Having such information would help build my confidence in caring for my newborn	
The information comes to me directly from the hospital	
I would know first-hand what to do and take appropriate action	

Frequency of messages

2. How often did you use/read the message that was sent? Read scale; tick only one]

Everyday	
Every alternate day	
Weekly	
Fortnightly	
Depending on the topic	
Any other? Specify	

3. Thinking about the frequency of the message how would you rate your experience? [Read scale; tick only one]

Highly dissatisfied	1
Dissatisfied	2
Satisfied	3
Highly satisfied	4
Don't know/not sure	5

If 1 or 2 coded above, ask how often would you like to receive the messages?

Every other day	
Weekly	
Fortnightly	
Monthly	
Any other? Please specify	

Satisfaction with the service

4. Overall, how satisfied were you with this service? [Read scale; tick only one]

Highly dissatisfied	1
Dissatisfied	2
Satisfied	3
Highly satisfied	4
Don't know/not sure	5

Service use

5. Thinking about the messages, how easy were the messages to understand?

Not at all easy	1
Not easy	2
Easy	3
Very easy	4
Don't know/not sure	5

Service experience

6. Which of the following statements best describes your experiences of the service [tick all that apply]?

The texts helped me to understand my situation and what I was going through	
It helped me to seek the right type of help at the right time	
I was able to handle many issues myself	
I trusted the information provided by the hospital	
I felt the hospital continued to care for me even after I was discharged	
It helped me to clarify conflicting information on topics like diet/activity/breastfeeding etc.	
It was information I could store and go back and read	
It reminded me about vaccination and review visits	
It addressed issues such as activity and contraception that I could then discuss with my doctor.	

7. Did you share the information with your friends and/or family?

Yes	If yes, what was the main purpose for sharing the information?
No	

8. Would you recommend this service to family and friends?

Yes	
No	

9. Currently there is no fee for this service. Would you be prepared to pay for this service?

Yes	
No	

10. Any other comments about the service? [Open ended question]

____

____

____

Collect Basic demographic information

11. Educational qualification:

Did not complete schooling	
Completed schooling	
Bachelor's Degree	
Above Bachelor's degree	
Any other?	

12. Are you employed? [Circle the correct response]

Yes	If yes, what is your current role?
No	

13. Which of the following describes your family unit?

Joint family [living with in-laws and/or the wider family]	
Nuclear family [husband/wife and their children]	
Any other	

14. Would you agree to being contacted for further research including potentially participating in a focus group to discuss your experiences in greater depth?

Verbal consent Yes/No [Circle their response]

Supplementary Table S2. Logistic regression results.

	B	S.E.	Wald	df	Sig.	Odds Ratio/Exp(B)	95% C.I. for EXP(B)	
							Lower	Upper
Age <25 years		2.842		2	0.242			
Age 25–35 years	−0.153	0.325	0.221	1	0.638	0.858	0.454	1.623
Age >35 years	0.137	0.286	0.228	1	0.633	1.146	0.655	2.008
Parity = 1		2.358		2	0.308			
Parity = 2	0.331	0.216	2.348	1	0.125	1.392	0.912	2.126
Parity > 3	0.264	0.222	1.413	1	0.235	1.302	0.843	2.013
No complications	−0.105	0.123	0.737	1	0.391	0.900	0.707	1.145
Type of delivery—Normal*	0.293	0.125	5.508	1	**0.019**	**1.341**	1.050	1.713
Constant	−0.078	0.323	0.058	1	0.810	0.925		

Bold: statistically significant.
